# Comparative Metabolomics Analysis of Citrus Varieties

**DOI:** 10.3390/foods10112826

**Published:** 2021-11-16

**Authors:** Dong-Shin Kim, Sun Lee, Suk Man Park, Su Hyun Yun, Han-Seung Gab, Sang Suk Kim, Hyun-Jin Kim

**Affiliations:** 1 Institute of Animal Medicine, Gyeongsang National University, Jinju 52828, Korea; feel567@naver.com; 2 Department of Food Science & Technology, Gyeongsang National University, Jinju 52828, Korea; pedric98@naver.com; 3 Citrus Research Institute, National Institute of Horticultural & Herbal Science, Rural Development Administration, Seogwipo 63607, Korea; babau2000@korea.kr (S.M.P.); yunsh@korea.kr (S.H.Y.); skhan@korea.kr (H.-S.G.); sskim0626@korea.kr (S.S.K.); 4 Division of Applied Life Sciences (BK21 Four), Institute of Agriculture and Life Science, Gyeongsang National University, Jinju 52828, Korea

**Keywords:** citrus varieties, GC-MS, HPLC, metabolomic, quality characteristics, UPLC-Q-TOF MS

## Abstract

Many citrus varieties are hybridized to improve their quality and to overcome the effects of climate change. However, there is limited information on the effect of the chemical profiles of hybrid varieties on their quality. In this study, we analyzed 10 citrus varieties and evaluated the correlation with their general characteristics and antioxidant activities. Chemical profiles, including the contents of sugars, organic acid compounds, flavonoids, limonoids, and carotenoids, which are related to taste, color, and health benefits, were significantly different depending on the citrus varieties, leading to different antioxidant capacities and general quality parameters. Based on these data, the correlations were investigated, and 10 citrus varieties were clustered into four groups—Changshou kumquat and Jeramon (cluster I); Setoka (cluster II-1); Natsumi, Satsuma mandarin, and Navel orange (cluster II-2); Kanpei, Tamnaneunbong, Saybyeolbong, and Shiranui (cluster II-3). Moreover, a metabolomic pathway was proposed. Although citrus peels were not analyzed and the sensory and functional qualities of the citrus varieties were not investigated in this study, our results are useful to better understand the relationship between citrus quality and metabolite profiles, which can provide basic information for the development and improvement of new citrus varieties.

## 1. Introduction

Citrus fruits are one of the most important fruits in the global food industry. They are mainly consumed as fresh fruits or as processed foods such as juices, jams, and purees, because they are rich in a variety of metabolites, including sugars, vitamins, flavonoids, limonoids, carotenoids, and dietary fiber [1,2,3]. Many clinical and animal studies have shown that some citrus metabolites can help protect against the effects of reactive oxygen species (ROS), improve digestive function, and prevent cardiovascular diseases, inflammation, diabetes, and neurological diseases [1,4,5]. Moreover, primary and secondary metabolites in citrus varieties are strongly associated with their unique qualities, such as taste, flavor, and appearance, as well as health benefits. The contents of these metabolites are generally affected by the cultivation environment, harvest period [6], and citrus variety [7]. Although there is a wide variety of citrus fruits, and these varieties have been extensively hybridized to improve their quality and to protect them against the effects of climate change [8,9], comparative analysis of metabolite profiles according citrus varieties has been limited. 

Recently, various technologies employed in the fields of genomics, proteomics, and metabolomics have been used to investigate the differences in the genetic, protein, and physicochemical profiles of citrus varieties [7,10,11]. Among these, nuclear magnetic resonance (NMR) and mass spectrometry (MS) have been used to compare the metabolite profiles of citrus varieties. Several metabolomic studies have been conducted on citrus varieties such as tangerines [12], grapefruits [6], mandarins, and oranges [7]. However, to date, the primary and secondary metabolites of kumquat, mandarin, and their hybrid varieties, which are cultivated as the major citrus varieties in East Asia [13], have not been compared, and the correlation between these metabolites and the quality characteristics of citrus fruits has not been investigated. 

Therefore, in this study, to better understand the correlation between the types and amounts of these metabolites and the quality characteristics of citrus varieties, flesh metabolite profiles of 10 citrus varieties were analyzed using gas chromatography–mass spectrometry (GC–MS), ultra-performance liquid chromatography–quadrupole-time-of-flight mass spectrometry (UPLC-Q-TOF-MS), and high-performance liquid chromatography (HPLC). Moreover, metabolomic pathways associated with citrus varieties have been proposed based on these metabolite profiles. The general characteristics of citrus fruits, including color, soluble solid content (SSC), pH, titratable acidity (TA), total flavonoid content (TFC), and antioxidant activities, were compared, and the correlation with the metabolite profiles was evaluated. 

## 2. Materials and Methods

### 2.1. Citrus Samples

**Figure 1:**
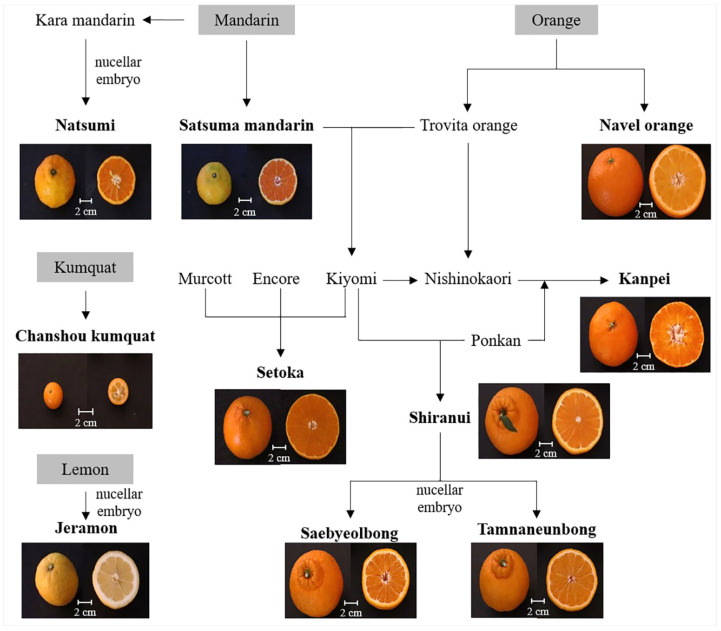
Appearance and parentage of 10 citrus varieties.

Among the 10 varieties (*n* = 5 per each variety), Changshou kumquat (*Fortunella obovata*) and Navel orange (*C. sinensis*) were obtained from a local market; the remaining eight, Satsuma mandarin (*C. unshiu*), Natsumi (*C. hybrid* cv. Natsumi), Jeramon (*C. limon* (L.) Burm. F.), Saebyeolbong (*C. reticulata* Blanco), Tamnaneunbong (*C. reticulata* Blanco), and Shiranui (*C. reticulata* cv. Shiranui), and kanpei (*C. hybrid* cv. Kanpei), and Setoka (*C. hybrid* cv. Setoka), were harvested from January 2017 to May 2017 and obtained from the Citrus Research Institute in Jeju, Korea. The parentage information of the 10 citrus varieties is presented in Figure 1. The diameter of each citrus variety was measured, and the flesh of each citrus variety was stored at −80 °C.

### 2.2. Color, SSC, pH, and TA

The flesh color values (L*, a*, and b*) of each variety were measured using a hand-held colorimeter (CR-400, Minolta, Tokyo, Japan), and the citrus color index (CCI) of flesh was calculated using the following formula [14]:

CCI = (1000 × a*)/(b* × L*).

The crushed flesh was used to measure SSC (in °Brix), pH, and TA. SSC of the flesh supernatant was measured using a handheld refractometer (Daihan Scientific Co., Wonju, Korea), and the pH was measured using a pH meter (Hanna Instruments, Woonsocket, RI, USA). TA was measured by preparing a solution of 1 mL supernatant, 1 mL distilled water, and 200 μL 1% phenolphthalein, and titrating it with 0.1 N NaOH solution till the endpoint. TA was expressed as percent citric acid in fresh weight. 

### 2.3. TFC and Antioxidant Activity

Lyophilized citrus flesh (0.05 g) was homogenized with 1 mL of 80% aqueous methanol using a bullet blender (Next Advance, Troy, NY, USA). After centrifugation, the supernatants were used to measure TFC and antioxidant activity. 

The TFC of the samples was measured using the AlCl_3_ method with minor modifications [15]. The sample (200 μL) was mixed with ethanol (800 μL) and 5% NaNO_2_ (60 μL). The reaction mixture was incubated at room temperature for 50 min, after which 10% AlCl_3_ (60 μL) was added and allowed to react with the rest of the reaction mixture for 6 min. After the reaction, 1 M NaOH (400 μL) and distilled water (400 μL) were added, and the absorbance was measured at 415 nm. Naringin was used as the standard compound, and TFC was expressed as mg naringin equivalents (NAR)/g dry matter (DM). 

The antioxidant activity of the samples was measured using four different methods: DPPH (2,2-Diphenyl-1-picrylhydrazyl) assay, ABTS (2,2′-azino-bis(3-ethylbenzothiazoline-6-sulfonic acid)) assay, ferric reducing antioxidant power (FRAP) assay, and hydrogen peroxide (H_2_O_2_) scavenging activity. DPPH radical scavenging activity was measured as described by Blois [16]. The sample (50 μL) was mixed with 0.1 mM DPPH solution (1 mL) and incubated at room temperature for 30 min in dark. After the reaction, the absorbance was measured at 520 nm. ABTS radical scavenging activity was measured as described by Re et al. [17]. The sample (20 μL) was mixed with 0.35 mM ABTS solution (980 μL) and incubated at room temperature for 6 min in dark. After the reaction, the absorbance was measured at 734 nm. FRAP scavenging activity was measured as described by Benzie and Strain [18]. The FRAP reagent was prepared by mixing 300 mM acetate buffer (pH 3.6), 10 mM 2,4,6-Tris(2-pyridyl)-s-triazine, and 20 mM FeCl_3_ in a ratio of 10:1:1 (*v*/*v*/*v*). The sample (50 μL) was mixed with distilled water (150 μL) and FRAP reagent (1.5 mL), and the reaction was allowed to occur for 30 min at 37 °C. After the reaction, the absorbance was measured at 520 nm. H_2_O_2_ scavenging activity was measured as described by Wettasinghe and Shahidi [19]. The sample (0.1 mL) was mixed with 0.3 mL of 40 mM H_2_O_2_ solution in 45 mM sodium phosphate buffer (pH 7.4) and incubated at room temperature for 40 min in dark. After the reaction, the absorbance was measured at 230 nm. The measured antioxidant activity was expressed as mg Trolox equivalents (mg TE)/g DM. 

### 2.4. Analysis of Metabolites Using GC-MS

A GC-MS-based citrus metabolite analysis was performed according to our previously described method with some modifications [20]. To extract the citrus flesh metabolites, the lyophilized sample was homogenized using a bullet blender (Next Advance, Troy, NY, USA) with 80% methanol containing dicyclohexyl phthalate as an internal standard (IS). After centrifugation, the supernatant was completely dried using a CentriVap Vacuum Concentrator (Labconco Co., Kansas City, MO, USA). The dried aliquot was dissolved in 70 μL pyridine containing methoxyamine hydrochloride (20 mg/mL) followed by incubation at 37 °C for 90 min and derivatization by the addition of 70 μL N,O-bis(trimethylsilyl)trifluoroacetamide with 1% trimethylchlorosilane at 70 °C for 30 min. The derivatized samples were analyzed using the GC-2010 plus system (Shimadzu Corp., Kyoto, Japan), equipped with a DB-5ms capillary column (30 m × 0.25 mm, 0.25 μm, Agilent J&W column) (Agilent Technologies, Santa Clara, CA, USA) with a split ratio of 1:50, a gas flow rate of 1 mL/min, and the injector temperature of 200 °C. The gradient program for oven temperature was as follows: maintained at 70 °C for 2 min; increased to 150 °C at a rate of 5 °C/min, to 210 °C at 3 °C/min, and to 320 °C at 8 °C/min; and finally held for 8 min at 320 °C. The eluted metabolites were detected using a GCMS-TQ 8030 MS (Shimadzu Corp., Kyoto, Japan) with electron ionization at 70 eV, ion source temperature of 230 °C, and interface temperature of 280 °C. The data were collected in the mass range of *m*/*z* 45–800. The quality control (QC) sample, prepared by mixing all the samples, was analyzed once between the sample sets. All MS data were normalized to the IS and identified by retention index (RI) values calculated with n-alkanes, authentic standards, and mass databases, including Wiley 9 and NIST 11.

### 2.5. Analysis of Metabolites Using UPLC-QTOF-MS

LC-MS-based citrus metabolite analysis was performed as per our previously described method, with some modifications [20]. To extract citrus flesh metabolites, the lyophilized sample was homogenized using a bullet blender with 80% methanol containing terfenadine as an IS. After centrifugation, the supernatants were used as metabolite extracts, which were analyzed using UPLC-Q-TOF-MS (Xevo^TM^ G2-S, Waters Corp., Milford, MA, USA). Both extracts were analyzed using an Acquity UPLC BEH C18 column (2.1 × 100 mm, 1.7 m; Waters Corp.) equilibrated with water containing 0.1% formic acid (FA). The metabolites, including carotenoids, eluted with a gradient of acetonitrile containing 0.1% FA were analyzed using the Q-TOF-MS with positive electrospray ionization (ESI) mode. source optimized as follows: a desolvation gas flow rate of 800 L/h, a desolvation temperature of 400 °C, an ion source temperature of 100 °C, a capillary voltage of 3 kV, and a sampling cone voltage of 40 V. The TOF-MS scan range was 100–1500 *m*/*z*, and leucine-enkephalin ([M + H] = 556.2771) was used as a lock mass to ensure accurate mass measurement of the metabolites. The MS/MS spectra were obtained using a collision energy ramp from 10 to 30 eV. Metabolite peaks were recorded over 1 s using the peak width at 5% height with an intensity threshold of 10,000, peak-to-peak baseline noise of one, and noise elimination set to six. Peaks were aligned within a 0.05 Da mass window and a retention time window of 0.2 min using MarkerLynx software (Waters Corp.). All mass spectra were normalized to the IS. The metabolites were identified based on online databases (Chemspider database in UNIFI and METLIN database (www.metlin.scripps.edu accessed on 19 August 2021)).

### 2.6. Analysis of Organic Acids, Vitamin C, and Carotenoids Using HPLC

For organic acid and vitamin C analysis, the lyophilized sample was homogenized with 5% aqueous meta-phosphoric acid using a bullet blender. After centrifugation, the supernatants were analyzed by HPLC (Shimadzu Corp., Kyoto, Japan) with a photodiode array detector (PDA; Shimadzu Corp., Kyoto, Japan). The extracted samples were injected into a Triart C18 column (250 × 4.6 mm, 4.6 mm I.D., 5 μm; YMC Co., Ltd., Kyoto, Japan.) and eluted with 0.1% phosphoric acid with isocratic elution at a flow rate of 1 mL/min and a column temperature of 40 °C. The eluted compounds were detected at 220 nm [21]. 

For carotenoid analysis, 0.25 g lyophilized sample was extracted using 5 mL of ethanol:hexane (4:3, *v*/*v*) with Na_2_CO_3_ (0.25 g) and 2,6-di-tert-butyl-methylphenol (BHT) (0.05 g) by shaking for 1 h at room temperature. After centrifugation, the supernatant was recovered, and the residue was re-extracted twice with 5 mL of ethanol:hexane (4:3, *v*/*v*). The combined supernatant was washed with 20 mL of distilled water and 20 mL of 10% NaCl and completely dried using a CentriVap Centrifugal Vacuum Concentrator. The dried residue was dissolved using methyl tert-butyl ether (MTBE), and the carotenoid extract was used. The extracts were analyzed using HPLC with a PDA detector. The extracted samples were injected into a carotenoid column (C30, 250 × 4.6 mm I.D., 5 μm; YMC Co. Ltd.) and eluted with solvent A (methanol:MTBE:water (81:15:4)) and solvent B (methanol:MTBE:water (6:90:4)) with gradient elution of solvent B from 0 to 66.6% for 60 min at a flow rate of 1 mL/min. The column temperature was 30 °C, and the eluted carotenoids were detected at 450 nm [22,23]. 

### 2.7. Statistical Analysis

Multivariate statistical analysis and hierarchical cluster analysis of experimental data were performed using SIMCA-P+ version 16.0.2 (Umetrics, Umeå, Sweden). Partial least-squares discriminant analysis (PLS-DA) was used to visualize the differences in the sample groups based on the metabolites analyzed using GC-MS, LC-MS, and HPLC. The PLS biplot was used to evaluate relationships and correlations between the metabolites and general characteristics (color, SSC, pH, TA, TFC, and antioxidant activity) of the citrus varieties. The differences between the experimental data were analyzed using one-way analysis of variance (ANOVA) with Duncan’s test (*p* < 0.05) using SPSS 24.0 (SPSS Inc., Chicago, IL, USA).

## 3. Results and Discussion

### 3.1. Characteristics of Citrus Varieties

The fruits of the 10 citrus varieties were classified by size, peel thickness, shape, and color (Figure 1). The diameter of Changshou kumquat (approximately 2 cm) was the smallest, while that of Satsuma mandarin, Natsumi, and Jeramon was approximately 6 cm, and that of the other varieties was more than approximately 8.5 cm. The peel thicknesses of the fruits of Setoka and Kanpei were relatively less compared to those of the other varieties. Shiranui, Tamnaneunbong, and Saebyeolbong had peak-shaped taps, unlike the other varieties. The flesh color of Jeramon was light yellow or light orange-yellow, while that of the other varieties was orange. 

To clearly compare the flesh color of the fruit varieties, the color values (L*, a*, and b*) and CCI of the flesh were measured (Table 1). Most varieties showed L* values in the range of 50.82–65.06, while those of the Changshou kumquat and Jeramon were more than 75. The a* values of most varieties were positive (0.47–18.45), but Jeramon and Changshou kumquat showed negative values (−3.74 to −1.29). The b* values of Satsuma mandarin, Navel orange, and Natsumi (42.41–47.84) were higher than those of the other varieties, but Jeramon, Tamnaneunbong, and Kanpei showed the lowest b* values (25.55, 29.57, and 28.70, respectively). Moreover, Natsumi showed the highest CCI (8.75), followed by Kanpei (6.21), Setoka (5.71), Saebyeolbong (4.70), Tamnaneunbong (4.21), Satsuma mandarin (3.32), and Navel orange (1.61), whereas Chanshou kumquat and Jeramon showed negative CCI (≤−0.49). These color differences between citrus varieties were associated with the carotenoid content—the higher the CCI value, the more orange it was, which had a significant correlation with the carotenoid content [24].

Jeramon had the lowest SSC value (7.24 °Brix), whereas the values of Satsuma mandarin and Natsumi were 11.16 and 12.38 °Brix, respectively, and those of the other citrus varieties ranged from 18.24 to 20.14 °Brix (Table 1). Citrus varieties had similar pH (3.52–4.22) and TA values (0.71–2.12%) except for Jeramon (pH, 2.67; TA, 5.06%). Based on the SSC and TA values, the sugar/acid ratio of citrus flesh were calculated, which is mainly used to determine maturity or taste parameters for citrus [25,26]. Saebyeolbong (14.31), Natsumi (16.00), and Kanpei (18.07) had higher sugar/acid ratios than the other varieties (8.36–12.85). In general, maturity indices are set at a sugar/acid ratio of at least 7.5 and 8 for mandarins and oranges, respectively. Citrus fruits with sugar/acid ratios of more than 14 are considered to be of good eating quality, and those with sugar/acid ratios higher than 20–22 are considered to be of flat taste [14]. Conversely, Jeramon had the lowest sugar/acid ratio (1.43) because it has a nucellar embryo of lemons that excessively accumulates citric acid during fruit development [25], and the sugar/acid ratio is not considered for lemons [14].

### 3.2. TFC and Antioxidant Activity

TFC of Setoka was the highest (5.18 mg NAR/g DM), while that of the other varieties ranged from 3.27 to 4.89 mg NAR/g DM (Table 1). Changshou kumquat showed the lowest TFC (3.27 mg NAR/g DM). 

Antioxidant activity of citrus varieties was determined in terms of DPPH, ABTS, FRAP, and H_2_O_2_ scavenging activities (Table 1). Jeramon and Setoka showed stronger scavenging activities for DPPH (3.99 and 3.93 mg TE/g DM, respectively) and ABTS (5.54 and 6.12 mg TE/g DM, respectively) than those by other varieties (DPPH: 2.04–2.60 mg TE/g DM and ABTS: 2.32–4.11 mg TE/g DM). Setoka also showed a strong FRAP (8.15 mg TE/g DM); FRAP of other varieties except Jeramon ranged from 4.65 to 5.06 mg TE/g DM; and Jeramon showed the lowest FRAP (2.11 mg TE/g DM), which was more than 2.2 times lower than other varieties. Most varieties had H_2_O_2_ scavenging activity in the range of 36.82–54.73 mg TE/g DM H_2_O_2_; however, Saebyeolbong had the lowest activity (29.58 mg TE/g DM). A previous report indicated that the pulp of Setoka (Cheonhyehyang) showed stronger DPPH radical scavenging activity and FRAP than the other citrus varieties, including Orange, Yuzu, Kanpei (J-Redhyang), Tangerin, and Shiranui (Hallabong), and that the antioxidant potential of citrus fruits is related to citrus antioxidants such as vitamin C, flavonoids, and tocols [27].

### 3.3. Metabolomic Analysis

**Figure 2:**
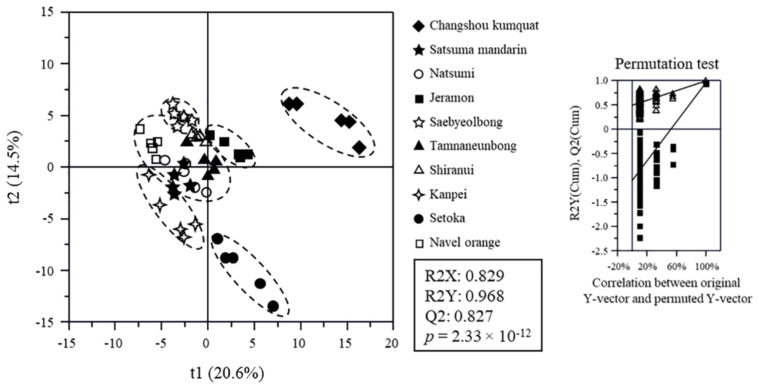
Partial least-squares discriminant analysis (PLS-DA) score plot of citrus metabolites analyzed using gas chromatography-mass spectrometry, ultra-high performance liquid chromatography-quadrupole time-of-flight mass spectrometry, and high-performance liquid chromatography with its qualifying parameters. The qualification of the PLS-DA model was evaluated using R2X, R2Y, Q2, and *p*-value and validated using cross validation with a permutation test (*n* = 200).

The metabolite profiles of citrus flesh, analyzed using GC-MS, UPLC-QTOF-MS, or HPLC (Figure S1), were statistically compared, and the discrimination of citrus varieties was visualized by PLS-DA score plots (Figure 2) based on their datasets. The goodness of fit (R2X = 0.741–0.999; R2Y = 0.522–0.968), predictability (Q2 = 0.426–0.827), *p*-values (2.17 × 10^−36^–9.14 × 10^−9^) (Table S1), and the cross-validation determined by the permutation test of the PLS-DA models indicated that the models were statistically acceptable (Figure 2). Changshou kumquat and Setoka were clearly separated from the other varieties by t(1) and t(2) on the score plot of all datasets. The other varieties were also separated from each other on the score plots of all datasets (Figure S2). In particular, the score plot of the HPLC-analyzed dataset of organic acids showed that Kanpei, Setoka, and Jeramon were clearly separated from each other and the other varieties (Figure S3C), and that of carotenoids showed that Natsumi and Navel oranges were clearly separated (Figure S3D). 

### 3.4. Identification of Major Metabolites

The variable importance in the projection (VIP) and *p*-values of all normalized chromatogram intensities of the citrus metabolites were analyzed to identify the metabolites contributing to the differences among citrus varieties (Tables S2–S4). Among them, 50 metabolites that have higher VIP (VIP ≥ 0.73) and lower *p*-value (≤0.05) were identified as the major metabolites contributing to the differences in the groups on the PLS-DA score plots. These metabolites included sugars (xylose, fructose, glucose, sorbitol, myo-inositol, galactose, and sucrose), amino acids (proline, 4-amino butanoic acid, aspartic acid, arginine, and stachydrine), lipids (palmitic acid, stearic acid, oleanitrile, oleamide, and lysophosphatidylcholines (LPCs) [18:2, 16:0, and 18:1]), acidic compounds (carbamic acid, quinic acid, oxalic acid, tartaric acid, malic acid, vitamin C, acetic acid, and citric acid), flavonoids (saponarin, chrysoeriol-7-diglucoside, rhoifolin, narirutin, diosmin, margaritene, hesperidin, isomagaritene, fortunellin, and didymin), limonoids (zapoterin, xylogranatin K, nomilin, and limonin), and carotenoids (violaxanthin, lutein, zeaxanthin, and carotenoid derivatives). 

### 3.5. Metabolic Pathway and Relative Abundance of Identified Metabolites

**Figure 3:**
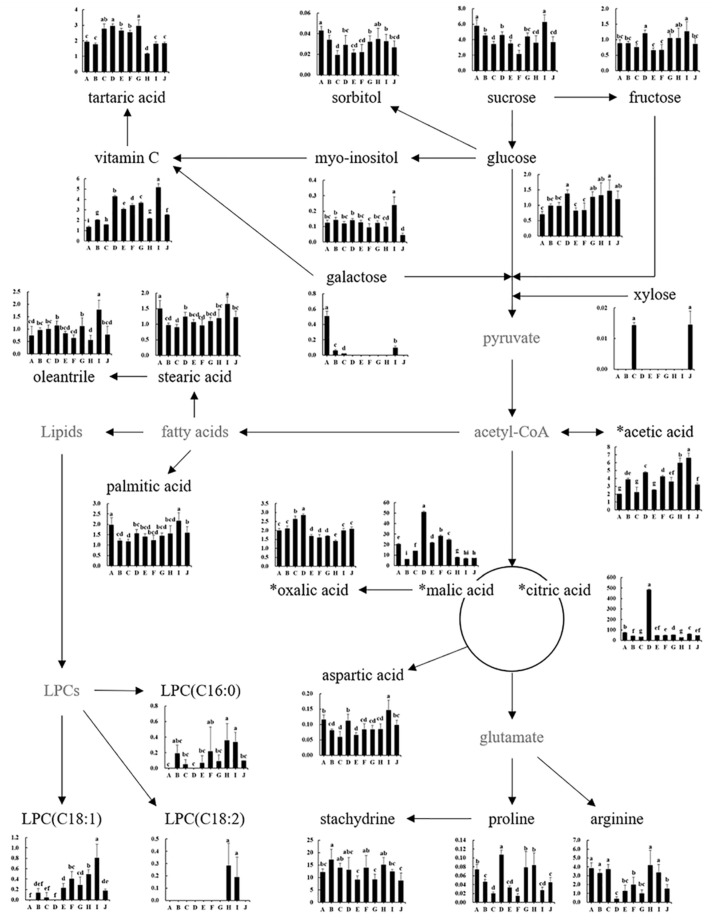
Schematic diagram of primary metabolomic pathway and relative abundance of citrus flesh metabolites. The *Y*-axis shows the normalized chromatogram intensity, and the *X*-axis shows citrus varieties. A, Changshou kumquat; B, Satsuma mandarin; C, Natsumi; D, Jeramon; E, Saebyeolbong; F, Tamnaneunbong; G, Shiranui; H, Kanpei; I, Setoka; J, Navel orange; LPC, lysophosphatidylcholine. * Organic acid compounds were quantitatively analyzed with authentic standards. The different letters on the bars indicate significant differences at *p* < 0.05.

**Figure 4:**
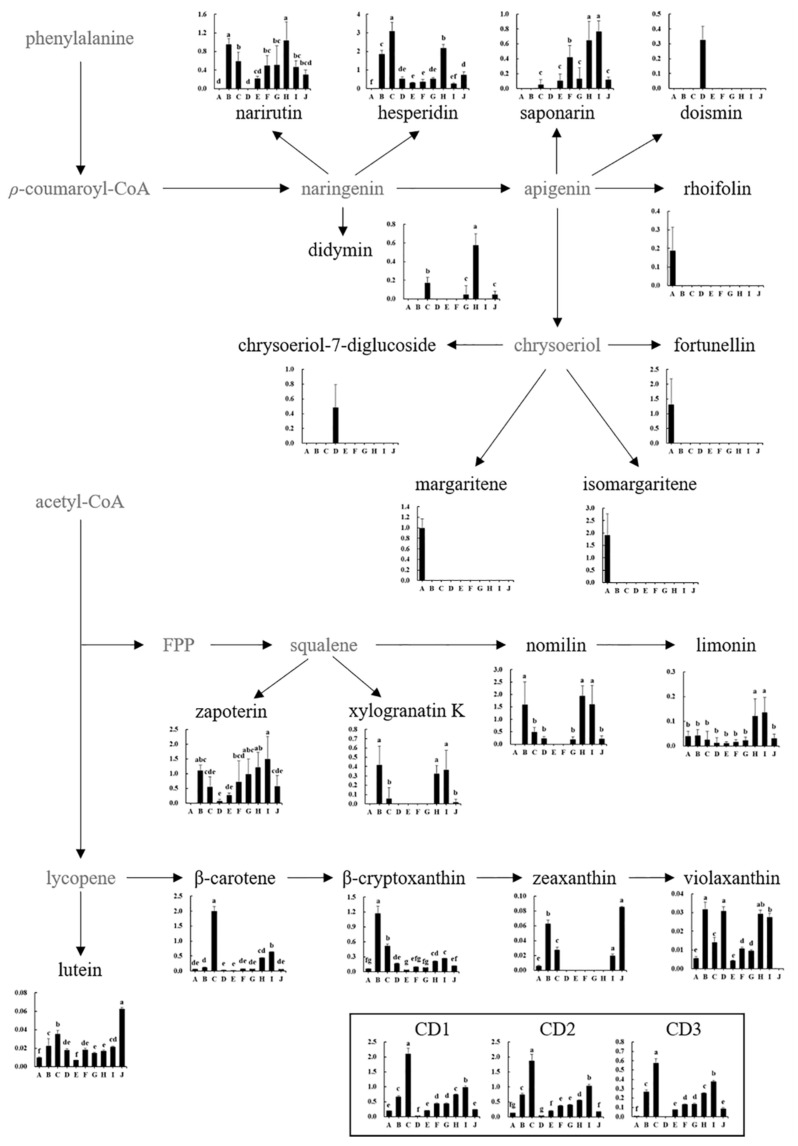
Schematic diagram of the secondary metabolomic pathway and relative abundance of citrus flesh metabolites. The *Y*-axis shows the normalized chromatogram intensity, and the *X*-axis shows citrus varieties. A, Changshou kumquat; B, Satsuma mandarin; C, Natsumi; D, Jeramon; E, Saebyeolbong; F, Tamnaneunbong; G, Shiranui; H, Kanpei; I, Setoka; J, Navel orange; FPP, farnesylpyrophosphate; CD, carotenoid derivatives. The different letters on the bars indicate significant differences at *p* < 0.05.

Based on the identified citrus flesh metabolites, the citrus metabolomic pathway was proposed, and the relative abundances of the metabolites were compared (Figure 3 and Figure 4). Sucrose, fructose, and glucose were identified as the major citrus sugars in the 10 varieties (Figure 3). The sum of the three main sugar amounts was the highest in Setoka, followed by Changshou kumquat, Jeramon, Satsuma mandarin, Shiranui, Kanpei, Navel orange, Natsumi, Saebyeolbong, and Tamnaneunbong, and the amount of main sugar in them was about 18–60% lower than that of Setoka. In addition, the ratio of sucrose:fructose:glucose in the 10 varieties was 3–5:1:1. However, xylose and galactose (minor sugars) have been observed in only a few varieties; xylose in Navel orange and Natsumi, and galactose in Satsuma mandarin, Natsumi, Setoka, and Changshou kumquat. In particular, the amount of galactose in Changshou kumquat, which was similar to the amount of glucose, was 8 to 24 times higher than that of Satsuma mandarin, Natsumi, and Setoka (Figure 3).

Among organic acids, citric acid was the main organic acid in citrus flesh, followed by malic acid, acetic acid, and vitamin C (Figure 3). In particular, Jeramon had the highest citric acid (482 mg/g DM) and malic acid content (60 mg/g DM), which were approximately 7–17 times and 2–9 times higher, respectively, than the other varieties because it is a nucellar embryo variety of lemon, which excessively accumulates citric acid during fruit development [25]. Kanpei (28 mg/g DM) and Natsumi (15 mg/g DM) had the lowest citric acid contents, while the lowest malic acid content was observed in Satsuma mandarin, Navel orange, Kanpei, and Setoka (approximately 6 to 8 mg/g DM). Setoka and Kanpei had the highest acetic acid content (about 6 mg/g DM), and the acetic acid content of the other varieties was approximately 2 to 4.8 mg/g DM. In particular, a high content of vitamin C, which has the strongest antioxidant activity in citrus [28], was observed in Setoka (5.1 mg/g DM) and Jeramon (4.3 mg/g DM), resulting in these varieties having the highest antioxidant activity (Table 1). Conversely, the vitamin C content of the other varieties was approximately 1.3 to 3.6 mg/g DM. Along with the sugar content, organic acids and their ratios are mainly evaluated to determine the maturity and major taste parameters in citrus fruits. In general, sugars accumulate, whereas organic acid content declines during fruit development, resulting in a sweet taste [25,26].

Flavonoids, limonoids, and carotenoids are the main secondary metabolites in citrus fruits and have various health benefits [1,4,5,29]. Among flavonoids, hesperidin and narirutin, which are major citrus flavonoids, were observed in most varieties, except in Jeramon and Changshou kumquat (Figure 4). Natsumi, Kanpei, and Satsuma mandarin had high amounts of hesperidin and narirutin, which were approximately 2.5 to 12.6 and 4.5 times higher than the other varieties, respectively. However, hesperidin was not observed in Changshou kumquat, whereas narirutin was not observed in either Jeramon or Changshou kumquat. Didymin was not observed or present in very low levels in all varieties except Kanpei and Natsumi. Interestingly, diosmin and chrysoeriol-7-diglucoside were found only in Jeramon, while rhoifolin and chrysoeriol derivatives (fortunellin, margaritene, and isomargaritene) were found only in Changshou kumquat (Figure 4). Flavonoid profiles are also related to citrus taste [30]. In general, most natural flavonoids are bitter and astringent in plants [31], but their taste differences depend on their structure [32]. Major citrus flavonoids, hesperidin and narirutin, have been reported to be tasteless; however, neohesperidin and naringin, which are structurally similar to hesperidin and narirutin, respectively, have a strong bitter taste [33,34]. Moreover, neohesperidin dihydrochalcone is extremely sweet, and some citrus flavonoids act as bitterness inhibitors [34,35]. Although the sensory quality characteristics between individual flavonoids and varieties were not evaluated in this study, differences in flavonoid profiles may also contribute to differences in taste between citrus varieties along with the sugar/acid ratios.

Among the 10 citrus varieties, zapoterin and nomilin were the main limonoids. Satsuma mandarin, Kanpei, Shiranui, and Setoka showed relatively higher contents of zapoterin (3.3 to 11.1 times higher than other varieties). In contrast, Jeramon, Saebyeolbong, and Tamnaneunbong showed relatively lower limonoid content, whereas Changshou kumquat only had limonin (Figure 4). Limonoids also contribute to citrus bitterness like flavonoids [36].

Among carotenoids, which are important pigments in citrus [37], Natsumi had the highest total carotenoid content (2 to 93 times higher than that in other varieties). Satsuma mandarin and Setoka also had a relatively high total carotenoid content compared with that in other varieties. β-cryptoxanthin, β-carotene, and carotenoid derivatives were the major carotenoids, and violaxanthin, lutein, and zeaxanthin were the minor carotenoids. Navel orange and peak-shaped tap citrus varieties had an orange color with low total carotenoid content compared to that in the orange-colored varieties. Additionally, Changshou kumquat and Jeramon had low total carotenoid contents (Figure 1 and Figure 4). 

### 3.6. Correlation between Quality Characteristics, Metabolites, and Citrus Varieties

**Figure 5:**
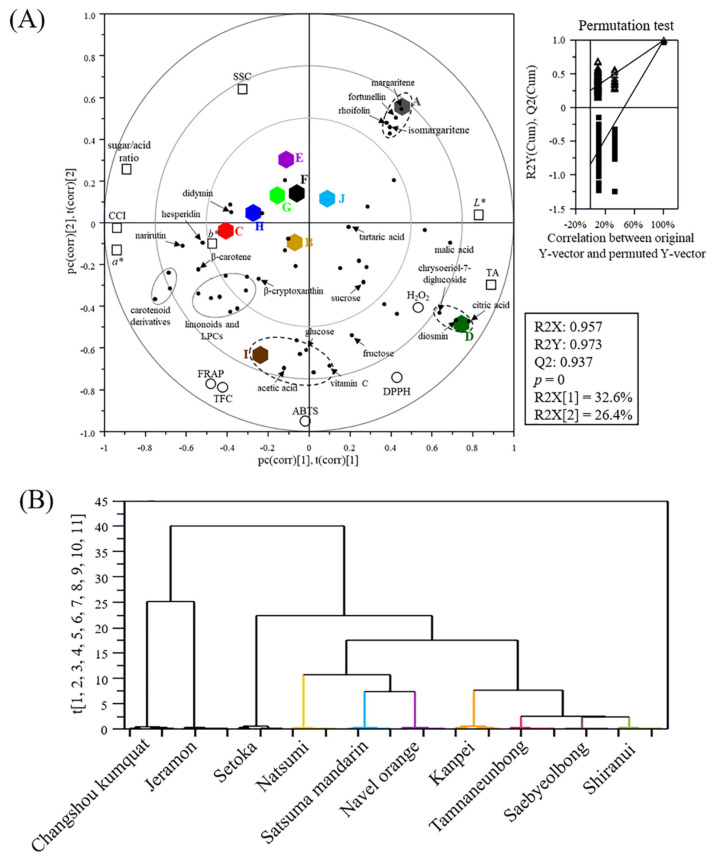
PLS-DA biplot (**A**) and hierarchical cluster analysis (**B**) of 10 citrus varieties based on the general quality parameters, antioxidant activities, and metabolite profiles. The qualification of the PLS-DA biplot was evaluated using R2X, R2Y, Q2, and *p*-values and validated using cross validation with a permutation test (*n* = 200). A, Changshou kumquat; B, Satsuma mandarin; C, Natsumi; D, Jeramon; E, Saebyeolbong; F, Tamnaneunbong; G, Shiranui; H, Kanpei; I, Setoka; J, Navel orange; L*, lightness, a*, redness, b*, yellowness.

The correlation was visualized, and correlation coefficients (r) were calculated (Tables S5 and S6) based on the results of quality characteristics and metabolite profiles of 10 citrus varieties (Figure 5A). The goodness of fit (R2X = 0.957; R2Y = 0.973), predictability (Q2 = 0.937), *p*-values (0), and cross-validation determined by the permutation test indicated that the PLS biplot was statistically significant. The PLS biplot indicated that there was a separation of three varieties (Changshou kumquat, Jeramon, and Setoka) from other varieties based on their specific metabolites (vitamin C, acetic acid, apigenin derivatives, and chrysoeriol derivatives). 

For citrus quality characteristics, the 10 varieties showed a wide range of SSC (7.24–20.14 °Brix) (Table 1), but there were no correlations between major sugars (sucrose, fructose, and glucose) and SSC (r = −0.23 to −0.07), because there were many factors that interfered with °Brix measurements, such as pectin [38]. Unlike sugars, major acidic compounds such as citric acid and malic acid showed strong correlations with TA (r ≥ 0.83) and sugar/acid ratio (r ≤ −0.67). Moreover, citric acid, malic acid, acetic acid, and vitamin C contributed to the unique qualities of Setoka and Jeramon, which are separated from other varieties on the biplot (Figure 5A). These results indicate that organic acid compounds are more important quality and metabolite factors than sugar in determining the sugar/acid ratio of citrus varieties because most citrus varieties have similar sugar content.

In addition, vitamin C positively contributed to citrus antioxidant activities determined by DPPH, ABTS, and FRAP assays, which showed a positive correlation (r ≥ 0.71). Setoka, which showed the highest content of vitamin C and strong antioxidant activity, was separated by pc(2) from other varieties (Figure 5A). In contrast, major secondary metabolites such as hesperidin and narirutin showed no correlation with antioxidant activities because the activity of vitamin C was much stronger than that of the secondary metabolites, including neohesperidin and naringin. Similar results were reported for Wando Yuzus and Maltease orange peel, where the antioxidant activity of vitamin C, as determined by ABTS and DPPH assays, was 7–9 and 21–22 times higher than that of hesperidin and naringin, respectively [39,40].

Most orange-colored varieties, some color values (a*, b*, and CCI), and carotenoids were separated by pc(1) from Chanshou kumquat and Jeramon (Figure 5A). In addition, β-cryptoxanthin (chief color of many orange-fleshed citrus fruits) [41] showed a positive correlation with b* (0.67) and CCI (0.61), while carotenoid derivatives showed a relatively high correlation with a* (r = 0.76–0.82) and CCI (r = 0.68–0.73). In general, carotenoid content and carotenoid structure are closely related to color, and their color depend on the number of double bonds in their polyene tail—β-cryptoxanthin (yellow), β-carotene (orange), lutein (orange), zeaxanthin (orange), and lycopene (red) [24,41].

### 3.7. Classification of Citrus Varieties

Based on the biplot, the 10 citrus varieties were classified using hierarchical cluster analysis and visualized using a dendrogram (Figure 5B). The 10 citrus varieties could be well categorized according to the differences in their quality characteristics and metabolite profiles: Changshou kumquat and Jeramon (Cluster I), and the other varieties (Cluster II). In fact, Changshou kumquat and Jeramon, which had been developed from Kumquat and Lemon, respectively, shared no parentage with the other varieties (Figure 1), and showed distinct differences in the general characteristics and metabolite profiles when compared with the other varieties. Cluster II was classified into three sub-clusters depending on parentage (Figure 1): Setoka, which was a hybrid of Kiyomi, Murcott, and Encore (II-1); Natsumi, Satsuma mandarin, and Navel orange (II-2); Kanpei, Tamnaneunbong, Saebyeolbong, and Shiranui, which was a hybrid of Kiyomi and Ponkan (II-3).

## 4. Conclusions

In this study, we analyzed the general characteristics, antioxidant activities, and metabolite profiles of the flesh of 10 citrus varieties, and their correlation was evaluated to compare the quality differences among them. Different citrus varieties showed significantly different chemical profiles (sugars, organic acid compounds, and secondary metabolites), which were related to citrus quality. The differences in chemical profiles were related to different antioxidant capacities and general citrus quality parameters such as SSC, pH, TA, and SSC/TA ratio. Based on the general quality parameters, antioxidant activities, and metabolite profiles, the 10 citrus varieties were clustered into four groups: Changshou kumquat and Jeramon (cluster I); Setoka (cluster II-2); Natsumi, Satsuma mandarin, and Navel orange (cluster II-2); Kanpei, Tamnaneunbong, Saybyeolbong, and Shiranui (cluster II-3). This clustering is very similar to the parentage differences. Moreover, a citrus metabolomic pathway has been proposed. Although the sensory and functional qualities of the 10 citrus varieties were not investigated, and their flavor was not analyzed in this study, these data are useful to better understand the relationship between citrus quality and metabolite profiles, which can provide basic information for the development and improvement of new citrus varieties.

## Figures and Tables

**Table 1:** General characteristics, total flavonoid content, and antioxidant activities of the flesh of citrus varieties.

	L*	a*	b*	CCI	SSC (°Brix)	pH	TA (%)	SSC/TA Ratio	TFC (mg NAR/g DM)	Antioxidant Activity (mg TE/g DM)			
										DPPH	ABTS	FRAP	H_2_O_2_
Changshou kumquat	86.62 ^a^	−1.29 ^i^	30.21 ^f^	−0.49 ^i^	20.14 ^a^	3.85 ^bc^	2.12 ^c^	9.53 ^f^	3.27 ^g^	2.44 ^c^	2.32 ^e^	2.11 ^e^	45.62 ^b^
Satsuma mandarin	75.58 ^c^	12.01 ^d^	47.84 ^a^	3.32 ^g^	11.16 ^e^	4.22 ^bcd^	0.96 ^e^	11.64 ^e^	4.73 ^bc^	2.04 ^e^	3.82 ^cd^	4.99 ^bc^	53.50 ^a^
Natsumi	50.82 ^h^	18.45 ^a^	42.41 ^b^	8.57 ^a^	12.38 ^d^	3.88 ^bcd^	0.77 ^f^	16.00 ^b^	4.89 ^b^	2.19 ^d^	4.00 ^c^	4.65 ^d^	43.34 ^c^
Jeramon	80.38 ^b^	−3.74 ^j^	25.17 ^h^	−1.85 ^j^	7.24 ^f^	4.05 ^f^	5.06 ^a^	1.43 ^h^	4.36 ^d^	3.99 ^a^	5.54 ^b^	4.65 ^d^	54.73 ^a^
Saebyeolbong	54.36 ^g^	8.22 ^f^	32.21 ^e^	4.70 ^e^	18.32 ^b^	3.80 ^e^	1.28 ^d^	14.31 ^c^	3.53 ^f^	2.40 ^c^	3.64 ^d^	4.81 ^bcd^	29.58 ^g^
Tamnaneunbong	54.95 ^g^	6.84 ^g^	29.57 ^f^	4.21 ^f^	19.06 ^b^	3.70 ^de^	1.66 ^c^	11.50 ^e^	4.26 ^d^	2.37 ^c^	3.86 ^cd^	4.73 ^cd^	38.68 ^e^
Shiranui	54.17 ^g^	14.39 ^b^	34.27 ^d^	7.75 ^b^	19.12 ^b^	3.67 ^de^	1.60 ^c^	11.93 ^de^	3.83 ^e^	2.60 ^b^	3.82 ^cd^	5.06 ^b^	36.82 ^f^
Kanpei	57.55 ^f^	10.26 ^e^	28.70 ^g^	6.21 ^c^	18.24 ^b^	3.69 ^ab^	1.01 ^e^	18.07 ^a^	4.48 ^cd^	2.15 ^de^	4.11 ^c^	5.06 ^b^	40.46 ^d^
Setoka	59.61 ^e^	12.87 ^c^	37.82 ^c^	5.71 ^d^	16.48 ^c^	3.59 ^cd^	1.28 ^d^	12.85 ^d^	5.18 ^a^	3.93 ^a^	6.12 ^a^	8.15 ^a^	42.23 ^c^
Navel orange	65.06 ^d^	4.47 ^h^	42.71 ^b^	1.61 ^h^	17.14 ^c^	3.91 ^a^	2.05 ^b^	8.36 ^g^	3.94 ^e^	2.21 ^d^	3.84 ^cd^	4.85 ^bcd^	46.47 ^b^

L*: lightness, a*: redness, b*: yellowness, CCI: citrus color index, SSC: soluble solids content, TA: titratable acidity, TFC: total flavonoid content, DM: dry matter, DPPH: 2,2-diphenyl-1-picrylhydrazyl, ABTS: 2,2′-Azino-bis(3-ethylbenzothiazoline-6-sulfonic acid) diammonium salt, FRAP: ferric reducing antioxidant power. Different letters in each column indicate significant differences by Duncan’s test (*p* < 0.05).

## Data Availability

Data is contained within the article and Supplementary Material.

## Supplementary Materials

The following are available online at https://www.mdpi.com/article/10.3390/foods10112826/s1, Figure S1: Representative chromatograms of citrus flesh metabolites analyzed by GC/MS (A), LC/MS (B), and HPLC (organic acid, C; carotenoids, D): Partial least-squares discriminant analysis (PLS-DA) score plots of citrus metabolites analyzed using GC/MS, UPLC-Q-TOF MS, and HPLC and their qualify parameters. The qualification of the PLS-DA models was evaluated using R2X, R2Y, Q2, and *p*-value and validated using cross validation with a permutation test (*n* = 200). (A) Satsuma mandarin, Natsumi, Jeramon, Saebyeolbong, Tamnaneunbong, Shiranui, Kampei, and Navel orange; (B) Satsuma mandarin, Natsumi, Saebyeolbong, Tamnaneunbong, Shiranui, Kampei, and Navel orange, Figure S2: Partial least-squares discriminant analysis (PLS-DA) score plot of citrus metabolites except for Changshou kumquat and Setoka (A) or Changshou kumquat, Jeramon, and Setoka (B) analyzed using GC/MS, UPLC-Q-TOF MS, and HPLC with its qualifying parameters. The qualification of the PLS-DA model was evaluated using R2X, R2Y, Q2, and *p*-value and validated using cross validation with a permutation test (n = 200), Figure S3: Partial least-squares discriminant analysis (PLS-DA) score plots of citrus flesh metabolites analyzed using GC/MS (A), UPLC-Q-TOF MS (B), and HPLC (organic acid, C; carotenoids, D) with its qualifying parameters., Table S1: Result of goodness of fit for each PLS-DA model, Table S2: Identification of major metabolites analyzed by GC-MS, Table S3: Identification of major metabolites analyzed by UPLC-Q-TOF MS, Table S4: Identification of major metabolites analyzed by HPLC, Table S5: Correlation between citrus quality characteristics and metabolites based on PLS-biplot, Table S6: Correlation between citrus varieties and citrus qualities, and metabolites based on PLS-biplot.
