# Supplementary material for: Comparative Metabolomics Analysis of Citrus Varieties
Source: Foods. 2021 Nov 16;10(11):2826. doi: 10.3390/foods10112826 (PMC8622604; doi:10.3390/foods10112826)
Supplement: Supplementary file 1 [file foods-10-02826-s001.zip › foods-1441778-supplementary-final-1119.pdf]

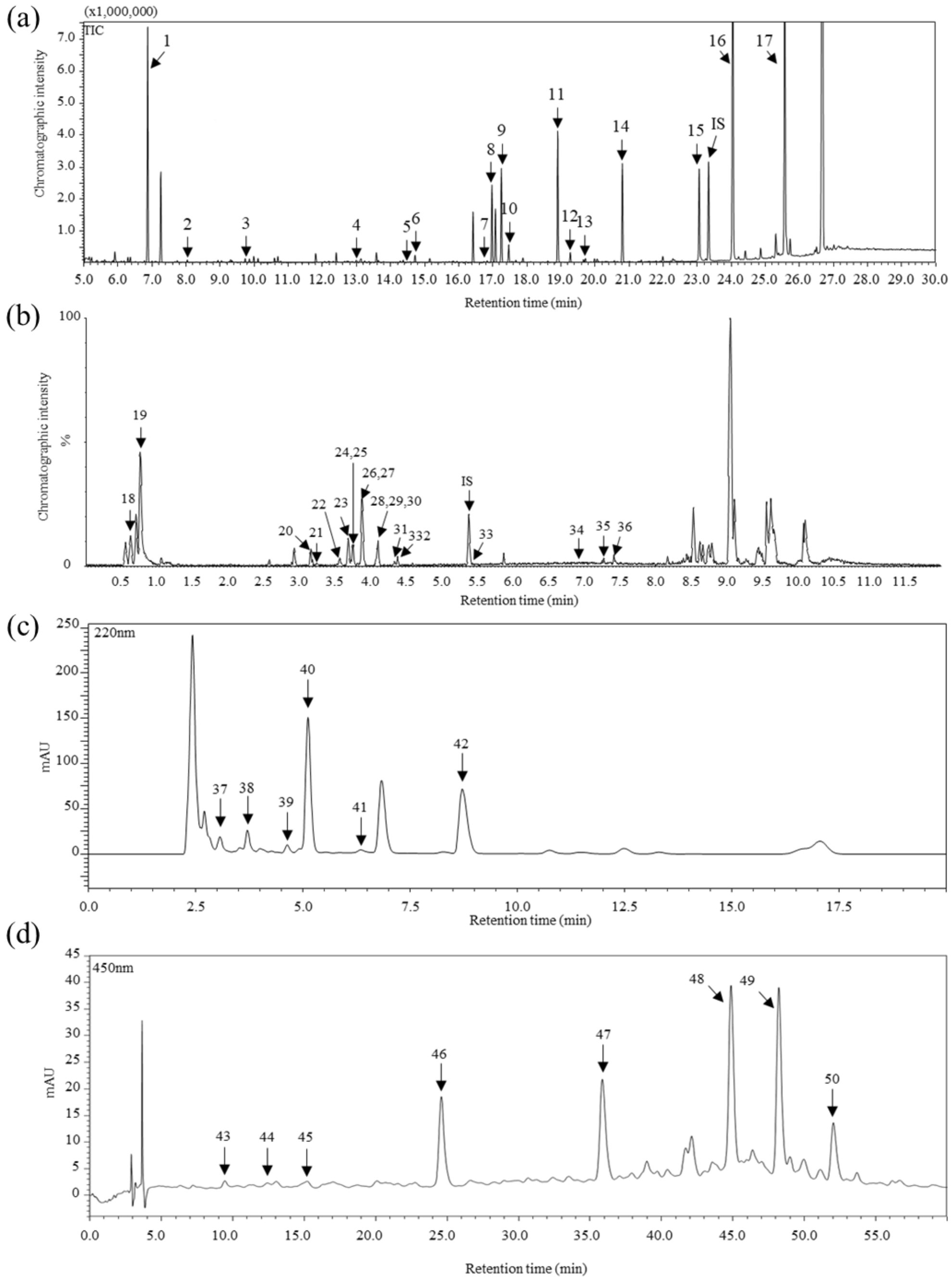

Figure S1. Representative chromatograms of citrus flesh metabolites analyzed by GC/MS (a), LC/MS (b), and HPLC (c and d). 1, 4,8-dimethylnonane; 2, carbamic acid; 3, proline; 4, 4-aminobutanoic acid; 5, xylose; 6, aspartic acid; 7, quinic acid; 8, fructose; 9, glucose; 10, sorbitol; 11, palmitic acid; 12, myo-inositol; 13, galactose; 14, stearic acid; 15, oleanitrile; 16, sucrose; 17, oleamide; 18, arginine; 19, stachydrine; 20, saponarin; 21, chrysoeriol-7-diglucoside; 22, rhoifolin; 23, zapoterin; 24, narirutin; 25, diosmin; 26, margaritene; 27, hesperidin; 28, isomargaritene; 29, xylogranatin K; 30, nomilin; 31, fortunellin; 32, didymin; 33, limonin; 34, LPC(C18:2); 35, LPC(C16:0); 36, LPC(C18:1); 37, oxalic acid; 38, tartaric acid; 39, malic acid; 40, vitamin C; 41, acetic acid; 42, citric acid; 43, violaxanthin; 44, lutein; 45, zeaxanthin; 46, β-cryptoxanthin; 47, β-carotene; 48-50: carotenoid derivatives.

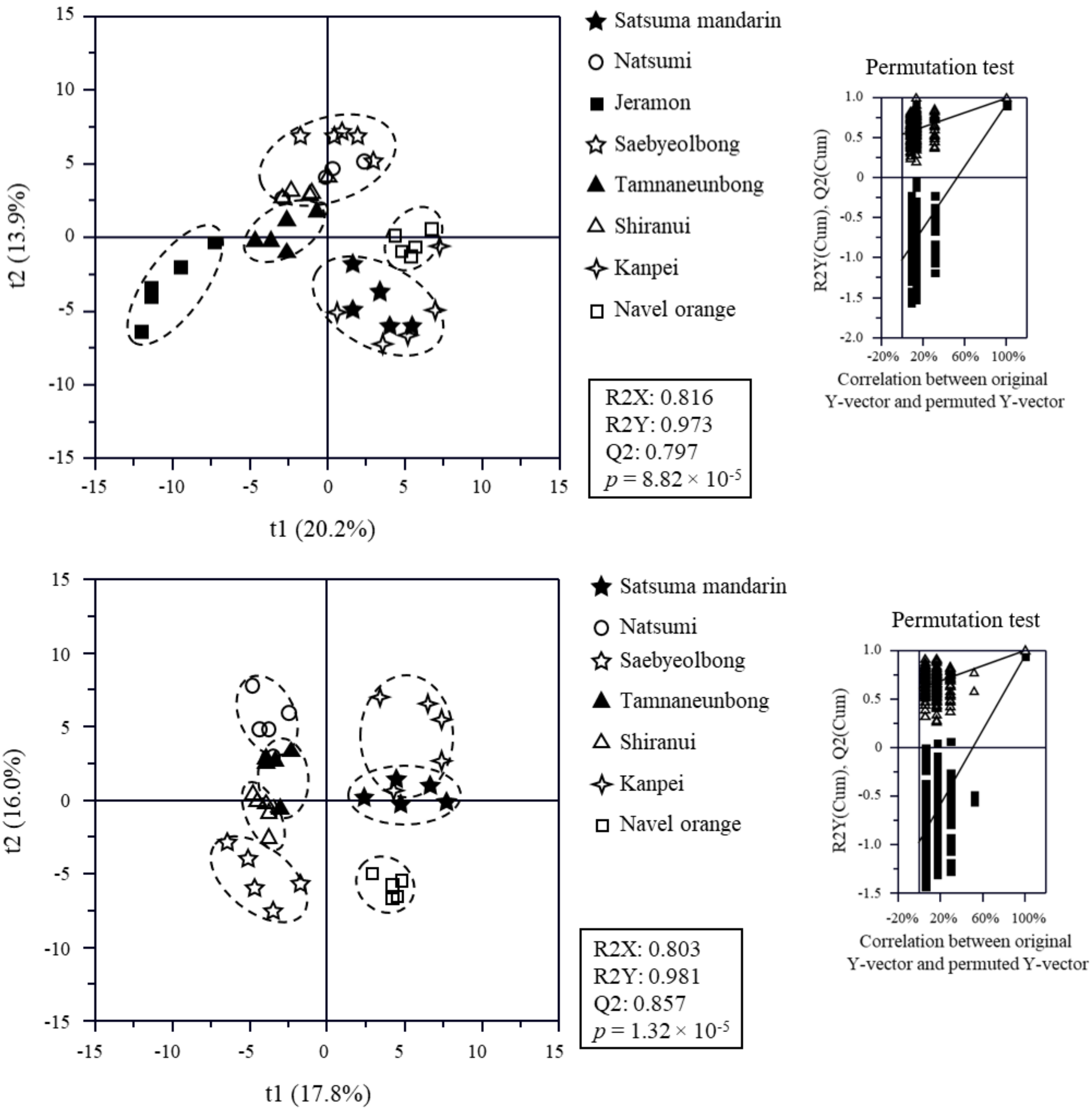

Figure S2. Partial least-squares discriminant analysis (PLS-DA) score plot of citrus metabolites except for Changshou kumquat and Setoka (A) or Changshou kumquat, Jeramon, and Setoka (B) analyzed using GC/MS, UPLC-Q-TOF MS, and HPLC with its qualifying parameters. The qualification of the PLS-DA model was evaluated using R2X, R2Y, Q2, and *p*-value and validated using cross validation with a permutation test (n = 200).

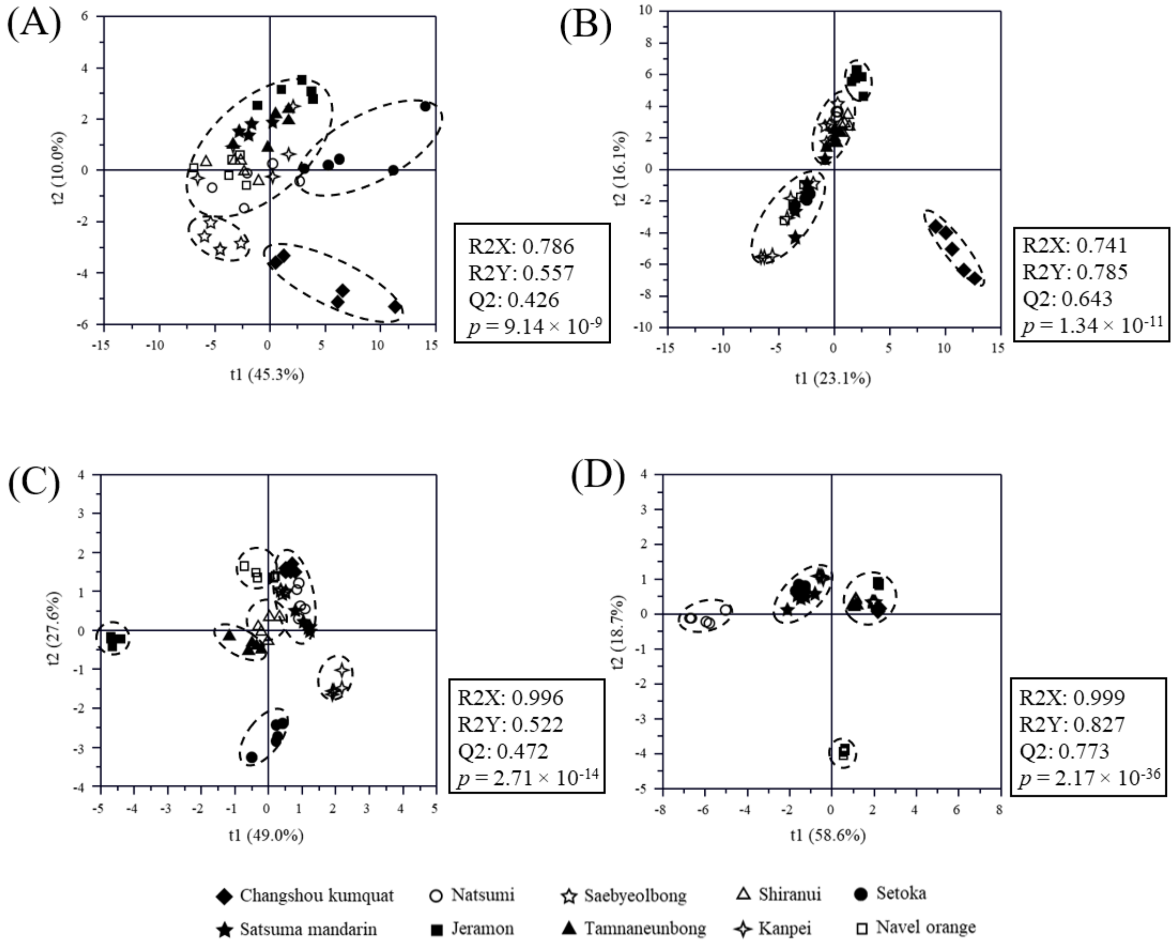

Figure S3. Partial least-squares discriminant analysis (PLS-DA) score plots of citrus flesh metabolites analyzed using GC/MS (A), UPLC-Q-TOF MS (B), and HPLC (organic acid, C; carotenoids, D) with its qualifying parameters. The qualification of the PLS-DA model was evaluated using R2X, R2Y, Q2, and p-value and validated using cross validation with a permutation test (n = 200).

Table S1. Quality parameters of PLS-DA models used to statically compare the discrimination of citrus

	PLS-DA models	R2X	R2Y	Q2	*p*-value
GC/MS, LC/MS, and HPLC	all varieties	0.829	0.968	0.827	2.23×10^{-12}
	all varieties except for Chansgshou kumuat and Setoka	0.816	0.973	0.797	8.82×10^{-5}
	all varieties except for Changshou kumuat, Jeramon, and Setoka	0.803	0.981	0.857	1.32×10^{-5}
GC/MS	all varieties	0.786	0.557	0.426	9.14×10^{-9}
LC/MS	all varieties	0.741	0.785	0.643	1.34×10^{-11}
HPLC	all varieties (organic acids)	0.996	0.522	0.472	2.71×10^{-14}
HPLC	all varieties (carotenoids)	0.999	0.827	0.773	2.17×10^{-36}

varieties

Table S2. Identification of major metabolites by GC-MS.

RT (min)	Compound	VIP	*p*-value	RI
6.8	4,8-dimethylnonane	0.81	7.65×10^{-6}	1098
8.0	carbamic acid	1.02	1.24×10^{-9}	1171
9.7	proline	1.41	1.12×10^{-10}	1281
12.9	4-aminobutanoic acid	1.10	4.04×10^{-12}	1513
14.5	xylose	1.28	1.04×10^{-12}	1637
14.7	aspartic acid	0.81	9.37×10^{-9}	1650
16.8	quinic acid	1.16	1.02×10^{-3}	1833
16.9	fructose	0.97	4.34×10^{-9}	1846
17.2	glucose	1.02	2.84×10^{-9}	1870
17.6	sorbitol	0.99	7.20×10^{-6}	1910
18.9	palmitic acid	0.81	1.36×10^{-6}	2028
19.2	myo-inositol	1.19	2.01×10^{-12}	2064
19.7	galactose	0.84	4.52×10^{-31}	2111
20.8	stearic acid	0.79	3.66×10^{-7}	2226
23.0	oleanitrile	1.11	1.21×10^{-6}	2485
24.0	sucrose	0.96	9.51×10^{-16}	2607
25.5	oleamide	0.86	9.35×10^{-4}	2809

RT, retention time; VIP, variable importance in the projection; RI, retention index.

p-values were analyzed by Duncan's test.

Table S3. Identification of major metabolites by UPLC-Q-TOF-MS.

RT (min)	Compound	VIP	*p*-value	Exact mass (m/z)	Fragment
0.64	arginine	0.98	5.48×10^{-7}	175.1223	
0.78	stachydrine	0.93	0.006	144.1035	
3.17	saponarin	1.13	1.10×10^{-8}	595.1616	433, 325
3.27	chrysoeriol-7-diglucoside	1.04	8.35×10^{-9}	625.1690	355, 409, 367, 379
3.49	rhoifolin	0.82	2.67×10^{-8}	579.1744	271, 433
3.69	zapoterin	0.99	5.24×10^{-7}	471.2009	317, 425
3.76	narirutin	1.00	4.50×10^{-10}	581.1932	273, 419
3.82	diosmin	1.13	2.73×10^{-17}	609.1871	301, 463, 286
3.85	margaritene	0.85	7.53×10^{-28}	593.1934	447, 429, 255
3.89	hesperidin	1.06	1.16×10^{-23}	611.1989	303, 449, 177
4.09	isomargaritene	0.73	2.18×10^{-8}	593.1952	327, 297, 579
4.10	xylogranatin K	1.04	1.37×10^{-8}	515.1946	455, 409
4.10	nomilin	0.93	7.05×10^{-10}	515.2308	
4.34	fortunellin	0.78	1.91×10^{-13}	593.1956	285
4.38	didymin	1.30	1.71×10^{-13}	595.2020	287, 433
5.43	limonin	0.88	7.73×10^{-7}	471.2020	161, 95
6.93	LPC(C18:2)	1.04	5.16×10^{-7}	520.3417	337, 460
7.27	LPC(C16:0)	0.82	1.26×10^{-6}	496.3445	184, 104
7.42	LPC(C18:1)	1.00	1.61×10^{-11}	522.3542	184, 104

RT, retention time; VIP, variable importance in the projection; LPC: lysophosphatidylcholine. *p*-values were analyzed by Duncan's test.

Table S4. Identification of major metabolites by HPLC.

	RT (min)	Compound	VIP	*p*-value	λ_{max} (nm)
Organic acids	3.06	oxalic acid	1.31	2.12×10^{-23}	
	3.53	tartaric acid	1.22	5.61×10^{-19}	
	4.63	malic acid	1.04	6.99×10^{-51}	
	5.12	vitamin C	1.11	1.11×10^{-36}	
	6.35	acetic acid	1.08	3.26×10^{-22}	
	8.72	citric acid	1.17	3.92×10^{-60}	
Carotenoids	9.1	violaxanthin	1.23	1.55×10^{-29}	440 , 471
	12.8	lutein	1.21	3.43×10^{-27}	420, 442
	15.0	zeaxanthin	1.24	2.66×10^{-43}	448, 432, 476
	24.6	β-cryptoxanthin	1.59	9.48×10^{-32}	448, 432, 476
	36.5	β-carotene	1.27	1.43×10^{-41}	426, 402, 451
	46.5	carotenoid derivatives 1	1.18	2.49×10^{-36}	448, 474
	50.3	carotenoid derivatives 2	1.17	3.32×10^{-33}	451, 469
	54.7	carotenoid derivatives 3	1.11	1.29×10^{-37}	448, 470

RT, retention time; VIP, variable importance in the projection. *p*-values were analyzed by Duncan's test.

Table S5. Correlation between citrus quality characteristics and metabolites based on PLS-biplot.

		SSC	TA	sugar/acid ratio	L*	a*	b*	CCI	TFC	DPPH	ABTS	FRAP	H_2O_2
	fructose	-0.23	0.31	-0.23	0.21	-0.10	-0.14	-0.12	0.23	0.55	0.52	0.35	0.27
	glucose	-0.24	0.22	-0.11	-0.10	0.08	-0.04	0.08	0.35	0.45	0.62	0.52	0.15
	sucrose	-0.07	0.15	-0.20	0.48	-0.15	0.06	-0.24	0.05	0.48	0.23	0.15	0.27
sugars	mannitol	0.12	0.08	-0.10	0.52	-0.25	-0.10	-0.28	-0.16	0.08	-0.16	-0.18	0.25
	myo-inositol	-0.19	-0.01	0.02	0.06	0.18	0.01	0.11	0.40	0.60	0.53	0.52	0.02
	galactose	0.30	0.03	-0.12	0.64	-0.40	-0.12	-0.42	-0.41	-0.02	-0.48	-0.54	0.16
	xylose	-0.14	-0.15	0.07	-0.24	0.24	0.52	0.17	0.12	-0.30	-0.08	-0.06	0.12
	arginine	0.17	-0.54	0.54	0.04	0.31	0.25	0.25	0.33	-0.30	-0.19	-0.01	0.07
	proline	-0.21	0.57	-0.40	0.48	-0.48	-0.46	-0.42	-0.28	0.24	0.02	-0.29	0.32
amino acids	aspartic acid	-0.02	0.32	-0.37	0.40	-0.35	-0.15	-0.38	0.06	0.61	0.38	0.27	0.27
	4-aminobutanoic acid	-0.02	0.09	-0.23	0.69	-0.33	-0.02	-0.43	-0.25	0.01	-0.32	-0.40	0.34
	stachydrine	-0.28	-0.08	0.10	0.15	0.10	0.10	0.01	0.43	-0.07	0.09	0.01	0.34
	oxalic acid	-0.79	0.54	-0.56	0.38	-0.21	0.17	-0.35	0.27	0.41	0.30	-0.07	0.63
	tartaric acid	-0.26	0.37	-0.35	-0.18	-0.02	-0.21	0.05	-0.18	0.23	0.05	-0.13	-0.16
	malic acid	-0.34	0.83	-0.67	0.26	-0.58	-0.70	-0.46	-0.28	0.49	0.15	-0.30	0.13
acids	vitamin C	-0.13	0.37	-0.32	-0.17	-0.06	-0.28	-0.01	0.28	0.81	0.78	0.71	-0.10
	acetic acid	-0.11	0.08	0.04	-0.12	0.07	-0.20	0.10	0.57	0.52	0.73	0.70	0.11
	citric acid	-0.68	0.95	-0.80	0.50	-0.64	-0.49	-0.64	0.02	0.69	0.46	-0.08	0.53
	carbamic acid	0.58	-0.25	0.22	-0.03	0.02	-0.04	0.09	-0.23	0.05	-0.11	0.06	-0.32
	quinic acid	-0.25	0.11	-0.18	0.22	-0.07	0.17	-0.15	0.11	0.26	0.16	0.04	0.26
	palmitic acid	0.18	0.16	-0.17	0.29	-0.27	-0.18	-0.25	-0.09	0.46	0.22	0.17	0.03
	stearic acid	0.14	0.20	-0.20	0.33	-0.30	-0.19	-0.29	-0.06	0.49	0.25	0.17	0.08
lipids	oleanitrile	-0.25	0.08	-0.13	-0.03	0.20	0.18	0.12	0.37	0.63	0.62	0.59	0.11
	LPC(C18:2)	0.17	-0.23	0.43	-0.17	0.19	-0.16	0.26	0.30	0.14	0.28	0.38	-0.08
	LPC(C16:0)	0.17	-0.34	0.38	-0.27	0.28	0.04	0.29	0.40	0.00	0.23	0.47	-0.14
	LPC(C18:1)	0.34	-0.35	0.39	-0.44	0.36	-0.04	0.42	0.36	0.25	0.44	0.73	-0.36
	saponarin	0.31	-0.30	0.41	-0.39	0.27	-0.17	0.36	0.44	0.24	0.44	0.63	-0.28
	chrysoeriol-7-diglucoside	-0.60	0.82	-0.66	0.39	-0.52	-0.41	-0.51	0.09	0.56	0.42	-0.06	0.44
	rhoifolin	0.31	0.07	-0.11	0.53	-0.41	-0.20	-0.39	-0.45	-0.07	-0.50	-0.59	0.10
	narirutin	-0.01	-0.56	0.56	-0.35	0.60	0.36	0.54	0.48	-0.39	0.03	0.30	0.00
	diosmin	-0.69	0.89	-0.74	0.44	-0.58	-0.46	-0.58	0.06	0.64	0.44	-0.06	0.52
flavonoids	margaritene	0.33	0.10	-0.16	0.62	-0.47	-0.23	-0.45	-0.53	-0.09	-0.57	-0.67	0.11
	hesperidin	-0.36	-0.42	0.50	-0.30	0.57	0.43	0.49	0.51	-0.44	-0.03	0.02	0.18
	isomargaritene	0.28	0.09	-0.15	0.54	-0.41	-0.19	-0.39	-0.46	-0.07	-0.49	-0.55	0.10
	fortunellin	0.31	0.09	-0.15	0.58	-0.44	-0.21	-0.42	-0.50	-0.07	-0.54	-0.60	0.11
	diymin	0.12	-0.29	0.55	-0.30	0.25	-0.17	0.37	0.18	-0.30	-0.01	0.02	-0.13
	capsanthin	-0.61	0.16	-0.05	0.20	0.09	-0.02	-0.02	0.69	0.41	0.62	0.42	0.51
	lutein	-0.16	-0.03	-0.13	-0.10	0.10	0.57	-0.02	0.22	-0.19	0.09	0.12	0.31
	zeaxanthin	-0.22	-0.19	-0.11	0.15	0.12	0.83	-0.11	0.20	-0.31	-0.04	0.10	0.45
	β-cryptoxanthin	-0.53	-0.32	0.14	0.17	0.40	0.71	0.13	0.53	-0.24	0.06	0.14	0.52
carotenoids	β-carotene	-0.24	-0.38	0.46	-0.42	0.62	0.35	0.57	0.55	-0.09	0.16	0.18	-0.01
	CD1	-0.18	-0.55	0.58	-0.49	0.76	0.47	0.70	0.64	-0.17	0.15	0.28	-0.03
	CD1	-0.23	-0.55	0.55	-0.46	0.78	0.53	0.68	0.69	-0.11	0.22	0.36	0.00
	CD1	-0.20	-0.61	0.61	-0.51	0.82	0.55	0.73	0.75	-0.12	0.28	0.46	-0.02
	zapoterin	0.12	-0.44	0.43	-0.33	0.52	0.29	0.47	0.52	0.00	0.30	0.58	-0.05
limonoids	xylogranatin K	-0.16	-0.37	0.33	0.04	0.36	0.36	0.22	0.56	0.03	0.32	0.48	0.24
	nomilin	-0.14	-0.35	0.40	-0.04	0.36	0.26	0.26	0.60	0.01	0.34	0.48	0.20
	limonin	0.15	-0.28	0.37	-0.08	0.24	0.02	0.24	0.38	0.18	0.31	0.46	-0.02

SSC: soluble solids content, TA: titratable acidity, CCI: citrus color index, TFC: total flavonoid content, DPPH: 2,2-diphenyl-1-picrylhydrazyl, ABTS: 2,2′-Azino-bis(3-ethylbenzothiazoline-6-sulfonic acid) diammonium salt, FRAP: ferric reducing antioxidant power, LPC: lysophosphatidylcholine, CD: carotenoid derivatives.

Table S6. Correlation between citrus varieties and citrus qualities, and metabolites based on PLS-biplot.

		A	B	C	D	E	F	G	H	I	J
general qualities	SSC	0.34	-0.39	-0.29	-0.71	0.20	0.26	0.26	0.19	0.05	0.10
	TA	0.10	-0.23	-0.29	0.93	-0.14	-0.03	-0.05	-0.22	-0.14	0.08
	sugar/acid ratio	-0.15	0.01	0.34	-0.77	0.21	0.00	0.03	0.49	0.10	-0.24
	L*	0.63	0.32	-0.36	0.46	-0.27	-0.25	-0.27	-0.18	-0.12	0.03
	a*	-0.48	0.19	0.52	-0.61	0.00	-0.07	0.31	0.10	0.23	-0.19
	b*	-0.23	0.61	0.35	-0.47	-0.14	-0.26	-0.04	-0.31	0.13	0.36
	CCI	-0.46	-0.07	0.47	-0.60	0.07	0.02	0.39	0.23	0.18	-0.24
antioxidant activities	TFC	-0.54	0.27	0.36	0.06	-0.39	0.01	-0.23	0.13	0.51	-0.17
	DPPH	-0.09	-0.29	-0.22	0.66	-0.11	-0.13	-0.02	-0.23	0.63	-0.20
	ABTS	-0.59	-0.10	-0.04	0.47	-0.15	-0.08	-0.09	0.00	0.66	-0.09
	FRAP	-0.68	0.02	-0.06	-0.06	-0.02	-0.04	0.04	0.04	0.79	-0.01
	H2O2	0.11	0.48	0.01	0.53	-0.62	-0.21	-0.29	-0.12	-0.04	0.15
sugars	fructose	-0.05	-0.06	-0.22	0.35	-0.34	-0.34	0.15	0.16	0.43	-0.08
	glucose	-0.40	-0.12	-0.12	0.29	-0.28	-0.26	0.18	0.23	0.38	0.10
	sucrose	0.42	0.09	-0.20	0.10	-0.18	-0.54	0.06	-0.15	0.54	-0.14
	mannitol	0.49	0.16	-0.37	-0.01	-0.29	-0.27	0.09	0.19	0.12	-0.10
	myo-inositol	0.00	0.11	-0.04	0.10	0.01	-0.20	-0.02	-0.17	0.74	-0.52
	galactose	0.97	-0.02	-0.11	-0.15	-0.15	-0.15	-0.15	-0.15	0.06	-0.15
	xylose	-0.16	-0.16	0.65	-0.16	-0.16	-0.16	-0.16	-0.16	-0.16	0.66
amino acids	arginine	0.31	0.19	0.29	-0.46	-0.27	-0.11	-0.33	0.39	0.20	-0.21
	proline	0.21	-0.07	-0.33	0.55	-0.20	-0.39	0.26	0.31	-0.26	-0.08
	aspartic acid	0.26	-0.13	-0.38	0.22	-0.31	-0.11	-0.10	-0.10	0.61	0.06
	4-aminobutanoic acid	0.69	0.39	-0.22	0.01	-0.19	-0.04	-0.12	-0.20	0.00	-0.33
	stachydrine	-0.03	0.41	0.12	0.05	-0.29	0.13	-0.28	0.22	0.00	-0.32
acids	oxalic acid	0.00	0.07	0.47	0.63	-0.24	-0.30	-0.23	-0.46	0.00	0.07
	tartaric acid	-0.17	-0.26	0.29	0.40	0.23	0.17	0.39	-0.59	-0.24	-0.22
	malic acid	0.04	-0.32	-0.12	0.81	0.08	0.24	0.15	-0.27	-0.30	-0.29
	vitamin C	-0.45	-0.25	-0.39	0.39	0.04	0.15	0.21	-0.22	0.64	-0.12
	acetic acid	-0.42	-0.01	-0.37	0.19	-0.30	0.08	-0.07	0.46	0.60	-0.16
	citric acid	-0.05	-0.12	-0.15	1.00	-0.11	-0.11	-0.10	-0.16	-0.08	-0.12
	carbamic acid	0.46	-0.42	-0.10	-0.43	-0.01	-0.21	0.09	0.05	0.42	0.16
	quinic acid	0.20	-0.07	0.27	0.11	-0.15	-0.25	-0.26	-0.25	0.28	0.12
lipids	palmitic acid	0.38	-0.27	-0.31	0.03	-0.11	-0.26	-0.07	0.01	0.55	0.06
	stearic acid	0.38	-0.25	-0.33	0.07	-0.14	-0.26	-0.10	0.02	0.56	0.05
	oleanitrile	-0.18	0.00	0.04	0.15	-0.10	-0.26	0.14	-0.32	0.68	-0.15
	LPC(C18:2)	-0.13	-0.13	-0.13	-0.13	-0.13	-0.13	-0.13	0.66	0.40	-0.13
	LPC(C16:0)	-0.27	0.09	-0.18	-0.27	-0.14	0.15	-0.10	0.41	0.37	-0.08
	LPC(C18:1)	-0.32	-0.15	-0.27	-0.32	-0.04	0.18	0.03	0.30	0.69	-0.10
flavonoids	saponarin	-0.25	-0.25	-0.20	-0.25	-0.14	0.22	-0.11	0.48	0.62	-0.12
	chrysoeriol-7-diglucoside	-0.09	-0.09	-0.09	0.85	-0.09	-0.09	-0.09	-0.09	-0.09	-0.09
	rhoifolin	0.84	-0.09	-0.09	-0.09	-0.09	-0.09	-0.09	-0.09	-0.09	-0.09
	narirutin	-0.39	0.43	0.11	-0.39	-0.21	0.04	0.05	0.50	0.00	-0.13
	diosmin	-0.11	-0.11	-0.11	0.97	-0.11	-0.11	-0.11	-0.11	-0.11	-0.11
	margaritene	0.99	-0.11	-0.11	-0.11	-0.11	-0.11	-0.11	-0.11	-0.11	-0.11
	hesperidin	-0.33	0.29	0.71	-0.16	-0.23	-0.21	-0.16	0.41	-0.25	-0.08
	isomargaritene	0.84	-0.09	-0.09	-0.09	-0.09	-0.09	-0.09	-0.09	-0.09	-0.09
	fortunellin	0.92	-0.10	-0.10	-0.10	-0.10	-0.10	-0.10	-0.10	-0.10	-0.10
	diymin	-0.16	-0.16	0.16	-0.16	-0.16	-0.16	-0.07	0.92	-0.16	-0.07
carotenoids	capsanthin	-0.31	0.44	-0.07	0.41	-0.34	-0.16	-0.19	0.37	0.31	-0.46
	lutein	-0.27	-0.01	0.28	-0.10	-0.34	-0.10	-0.17	-0.12	-0.03	0.86
	zeaxanthin	-0.17	0.49	0.08	-0.23	-0.23	-0.23	-0.23	-0.23	-0.01	0.75
	β-cryptoxanthin	-0.21	0.91	0.24	-0.11	-0.24	-0.18	-0.19	-0.06	0.00	-0.16
	β-carotene	-0.16	-0.13	0.94	-0.18	-0.20	-0.16	-0.16	0.05	0.16	-0.16
	CD1	-0.24	0.04	0.87	-0.33	-0.23	-0.10	-0.09	0.08	0.22	-0.21
	CD1	-0.27	0.12	0.83	-0.32	-0.22	-0.11	-0.09	0.00	0.30	-0.24
	CD1	-0.35	0.15	0.74	-0.37	-0.23	-0.12	-0.11	0.12	0.36	-0.20
limonoids	zapoterin	-0.37	0.22	-0.08	-0.34	-0.23	0.01	0.16	0.28	0.43	-0.07
	xylogranatin K	-0.20	0.52	-0.11	-0.20	-0.20	-0.20	-0.20	0.36	0.43	-0.18
	nomilin	-0.25	0.39	-0.05	-0.16	-0.25	-0.25	-0.18	0.54	0.40	-0.17
	limonin	-0.04	-0.02	-0.12	-0.21	-0.22	-0.19	-0.15	0.48	0.57	-0.09

A: Oval kumquat, B: Satsuma mandarin, C: Natsumi, D: Jeramon, E: Saebyeolbong, F: Tamnaneunbong, G: Shiranui, H: Kanpei, I: Setoka, J: Navel orange, SSC: soluble solids content, TA: titratable acidity, CCI: citrus color index, TFC: total flavonoid content, DPPH: 2,2-diphenyl-1-picrylhydrazyl, ABTS: 2,2′-Azino-bis(3-ethylbenzothiazoline-6-sulfonic acid) diammonium salt, FRAP: ferric reducing antioxidant power, LPC: lysophosphatidylcholine, CD: carotenoid derivatives
